# Integrated Child Development Services (ICDS) and their contribution to improving complementary feeding practices among children aged 6–23 months in India: insights from a nationally representative survey

**DOI:** 10.1186/s12889-025-25884-2

**Published:** 2025-12-11

**Authors:** R Praveen Kumar, Y Selvamani, Hari Singh

**Affiliations:** School of Public Health, Faculty of Medicine and Health Sciences, SRM Institute of Science and Technology, Kattankulathur, Chengalpattu, Tamil Nadu India

**Keywords:** Malnutrition, Stunting, Feeding practices, IYCF, Child development, ICDS

## Abstract

**Background:**

Integrated Child Development Services (ICDS) is India’s flagship programme to improve the nutritional and health status of children. Appropriate complementary feeding is essential for young children age 6 and 23 months to meet additional nutrient needs. However, there are limited evidence on how ICDS utilization affects complementary feeding practices in India.

**Methods:**

This study used a sample of 59,115 children age 6–23 months from the National Family Health Survey 2019-21. Modified Poisson regression analysis was performed to evaluate the association between the utilization of ICDS services and complementary feeding practices, estimating the adjusted prevalence ratio (aPR).

**Results:**

Children age 6–23 months receiving ICDS services had a 19% high probability of consuming diverse diet (95% CI 1.13–1.25, *p* < 0.001), a 27% high probability of consuming egg and flesh foods (95% CI 1.20–1.34, *p* < 0.001), and a 3% higher probability of consuming vegetables and fruits (95% CI 1.00–1.06, *p* < 0.05). However, ICDS utilization is not significantly associated with the timely introduction of semi-solid foods (6–8 months) and the minimum acceptable diet (6–23 months). The probability of achieving recommended meal frequency is significantly 14% lower (95% CI 0.83–0.90, *p* < 0.001) among ICDS beneficiaries.

**Conclusion:**

Despite the programme expansion, ICDS have limited influence on improving complementary feeding practices in India. Targeted efforts to improve service delivery to young children by Integrated Child Development Services could potentially support better complementary feeding practices.

## Background

From conception to two years of age, good nutrition during these first 1000 days of life is critically important for optimal growth and development, as infants and young children have greater nutrient needs per kilogram of body weight than older children do [1]. If these nutritional needs are not met through adequate breastfeeding and appropriate complementary feeding, infants are at increased risk of developing nutrient deficiencies and experiencing impaired growth and development, which can have long-lasting consequences. Globally, malnutrition is attributed to almost half of the deaths of children under 5 years of age. In addition to low birth weight being directly related to maternal malnutrition, children often develop stunting, wasting and underweight within the first few months of life hindering the healthy pace of growth and development [2]. India faces a significant malnutrition burden, with 35.5% under five children stunted, 19.3% wasted, and 32.1% underweight, according to the National Family Health Survey (NFHS-5, 2019–21) [3].

World Health Organization (WHO) 2023 guidelines on complementary feeding for children aged 6–23 months emphasizes the introduction of appropriate foods alongside breast milk to meet nutritional needs, promote healthy dietary patterns, and reduce the risk of growth faltering and nutrient deficiencies [4]. Appropriate complementary feeding involves introducing variety of foods at 6 months, progressively diversifying consistency and variety, and increasing the meal frequency to 2–4 times daily with snacks as needed, alongside continued breastfeeding [4]. Appropriate complementary feeding is identified among ten core nutrition interventions, if scaled to 90% in 34 focus countries accounting for 90% of the global burden of stunted growth, could lead to a 15% reduction in mortality in children younger than 5 years (saving approximately 1 million lives) and a 20.3% reduction in stunting (33.5 million fewer stunted children [5]. In the context of stunting prevention, complementary feeding is one of the central pillars that support a healthy pace of growth and development. In India, 23% of children aged 6–23 months receive the minimum dietary diversity, 35% meet the minimum meal frequency, and only 11% achieve the minimum acceptable diet [6]. Globally, the corresponding proportions are 29%, 53%, and 19%, respectively [7]. It is crucial to assess the various contextual determinants along the continuum of care to inform programs and policies that enhance child health and nutrition through a holistic approach.

India has two major programs—National Health Mission (NHM) and Integrated Child Development Services (ICDS)—that implement various interventions to improve maternal and child health. While the NHM focuses on preventive and curative health and family welfare services, the ICDS provides early childhood care programs addressing the malnutrition and development needs of children and mothers. Integrated Child Development Services (ICDS) was launched in 1975 under the Ministry of Women and Child Development with the aim of improving the health and nutritional status of children and women [8]. Anganwadi Services under the ICDS provides six interventions: supplementary nutrition, preschool nonformal education, nutrition and health education, immunization, health check-ups, and referral services for children under six years of age, pregnant and lactating women [9]. First three of these services are provided by ICDS through community-based workers referred to as Anganwadi workers (AWWs) and the remaining three services by NHM through referrals.

The effectiveness of the ICDS in reducing malnutrition could be closely linked to the promotion of optimal infant and young child feeding (IYCF) practices. Several studies have evaluated the impact of ICDS on anthropometric outcomes [10–12], and nutrient and calorie intake as well [13–15]. However, there is limited evidence on how ICDS utilization affects complementary feeding practices in India using a nationally representative survey. This study aimed to fulfil this gap by estimating the association between the ICDS utilization among young children and complementary feeding practices in India using national survey data.

## Methods

### Data source

This cross-sectional study utilized data from India’s National Family Health Survey (NFHS-5) 2019–2021. The NFHS was conducted by the Ministry of Health and Family Welfare (MoHFW) in collaboration with the International Institute for Population Sciences (IIPS), Mumbai, and ICF International under the Demographic and Health Survey (DHS) Program. The survey utilized a stratified two-stage sampling design implemented in rural and urban areas of 707 districts. A detailed survey methodology could be found elsewhere [6].

This study included a weighted sample of 59,115 lastborn children aged 6 to 23 months residing with their mothers for further analysis. In the survey, women were also asked to recall the variety and frequency of foods and liquids their youngest child consumed during the previous day and night. This includes recording the consumption of specific food groups, such as grains, fruits, vegetables, meats, and dairy products, as well as liquids such as milk and infant formula.

### Ethical approval

Ethical approval for the NFHS survey was provided by the International Institute for Population Sciences (IIPS), Mumbai. Informed consent from the respondents was obtained for their participation in the survey. Following registration and requests with the IIPS data website, we obtained authorization to use the datasets. The datasets from the NFHS-5 are anonymized and deidentified.

### Measures

#### Outcome measures

This study assessed six complementary feeding indicators based on the 2021 WHO UNICEF guidelines for assessing infant and young child feeding practices to calculate the proportion of children meeting the criteria [16].

**Table:** 

SNo	Complementary feeding indicators	Criteria
1	Introduction of Solid, Semisolid or Soft Foods (ISSSF)	Proportion of children age 6 to 8 months who consumed solid, semisolid or soft foods
2	Minimum Dietary Diversity (MDD)	Proportion of children age 6 to 23 months who consumed foods and beverages from at least five out of eight food groups. The eight food groups are: (1) breast milk, (2) grains, roots, and tubers, (3) pulses, nuts, and seeds, (4) dairy products, (5) flesh foods, (6) eggs, (7) vitamin A-rich fruits and vegetables, and (8) other fruits and vegetables.
3	Minimum Meal Frequency (MMF)	Proportion of children age 6–23 months who had consumed foods at least the minimum number of times the previous day. The minimum number of times are 2 feedings for breastfed infant age 6–8 months, 3 feedings for breastfed infant age 9–23 months, and 4 milk feeds and at least one non-milk feeds for non-breastfed infant age 6–23 months.
4	Minimum Acceptable Diet (MAD)	Proportion of children age 6–23 month who had consumed sufficient food variety and appropriate frequency. This indicator combines MDD and MMF with additional information on non-breast infants received milk at least the previous day.
5	Egg and/or Flesh Food consumption (EFF)	Proportion of children aged 6–23 months who consumed egg and/or flesh food from poultry, meat or fish
6	Vegetable or Fruits Consumption (VFC)	Proportion of children aged 6–23 months who consumed any vegetables or fruits, including vitamin A-rich fruits, dark green leafy vegetables and other fruits and vegetables

### Exposure variable

ICDS Utilization is coded “Yes” if the child utilized all three components from ICDS services - received take-home rations at least once a week, their weight measured at least once a month, and had health check-up at the Anganwadi centre at least once a month in the last 12 months.

### Covariates

Covariates were selected following existing literature and their availability in the dataset [17–19]. The child-related variables include age (6–11 months/12–18 months/19–23 months), sex (male/female), low birth weight, birth order (1st/2nd/3rd or higher), and recent illness. Low birth weight is coded “Yes” if the weight of the baby when born is less than 2500 g. Recent illness was coded as ‘Yes’ if the child experienced fever, cough, or diarrhea in the past two weeks. Maternal characteristics include age (15–24 years/25–34 years/35–49 years), education (no education/primary/secondary/higher), and exposure to mass media. Exposure to mass media is coded “Yes” if the woman read newspaper/magazine, listen radio or watch television at least once a week. Household characteristics consisted of place of residence (Urban/Rural), wealth quintile, religion (Hindu/Muslim/Christian/Others), and caste (Scheduled caste/Scheduled tribes/Backward castes/Unreserved). The wealth quintile variable was coded as poorest, poorer, middle, richer and richest. Wealth quintile was based on the household ownership of consumer goods, possessions, and characteristics of dwelling such as flooring materials, drinking water and toilet facilities. Wealth index score was derived using principal component analysis (PCA) and these wealth scores were divided into five equal groups [6]. Indian states and union territories were classified into six geographical regions (South/North/Central/East/West/Northeast).

### Statistical analysis

The study analyzed a sample of 59,115 children aged 6–23 months from the National Family Health Survey 2019–2021. Weighted descriptive statistics were used to account for the complex sampling design. The overall proportion of children who met the criteria for complementary feeding indicators was calculated. The association between the child’s utilization of ICDS services with each of the six complementary feeding indicators were estimated separately, controlling for the covariates. As the prevalence rate of outcomes was more than 10%, we used modified Poisson regression with robust variance to estimate the prevalence ratios, which yields more accurate estimates in cross-sectional surveys [20, 21]. The adjusted Prevalence Ratio (aPR) with 95% CI was reported. All analyses were conducted using Stata 17.0 (StataCorp LLC, College Station, TX, USA), accounting for the complex survey design. A significance level of 5% (α = 0.05) was used.

## Results

### Sociodemographic characteristics of the study participants

In the overall sample, 51.9% of children were male, 17.9% had low birth weight, and 28.7% reported recent illness (Table 1). Over half of the mothers were aged 25–34 years (52.5%), had secondary education (52.5%) and were exposed to mass media (51.1%). The majority of the children were from rural areas (73.1%), Hindu (79.2%), and backward caste (45.6%). Our analysis suggests that 30.5% of the children (95% CI: 29.8–31.1) were utilizing all three components from ICDS - receipt of take-home rations at least once a week, their weight measured at least once a month, and had a health check-up once a month at the Anganwadi centre in the last 12 months. Central (26.5%) and East regions (26.6%) contributed a higher proportion to the overall sample.

**Table 1:** Sociodemographic characteristics of children age 6–23 months assessed for complementary feeding practices in India, NFHS 2019−21 (*N* = 59,115)

Characteristics	ISSSF *n* (%)	Total *n* (%)	MDD *n* (%)	MMF *n* (%)	MAD *n* (%)	EFF *n* (%)	VFC *n* (%)	Total *n* (%)
N =	10, 185 (age 6 to 8 months)		59, 115 (age 6 to 23 months)					
Child’s age								
6 months	1,385 (26.6)	3,630 (35.7)	NA					
7 months	1,720 (34.1)	3,349 (33.0)						
8 months	1,987 (39.3)	3,206 (31.3)						
Child’s age group								
6–11 months	NA		2,770 (19.5)	5,060 (27.1)	1,189 (19.4)	2,558 (19.1)	6,239 (21.9)	20,338 (34.7)
12–17 months			5,758 (38.1)	7,306 (38.8)	2,723 (41.5)	5,313 (37.8)	11,385 (38.3)	20,742 (34.5)
18–23 months			6,007 (42.4)	6,105 (34.1)	2,411 (39.1)	5,639 (43.1)	11,403 (39.8)	18,035 (30.8)
Child’s sex								
Male	2,588 (51.2)	5,197 (50.6)	7,502 (52.0)	9,521 (51.7)	3,237 (51.5)	6,885 (51.6)	15,041 (52.1)	30,679 (51.9)
Female	2,504 (48.8)	4,988 (49.4)	7,033 (48.0)	8,950 (48.3)	3,086 (48.5)	6,625 (48.4)	13,986 (47.9)	28,436 (48.1)
Low birth weight								
No	3,912 (82.8)	7,676 (81.9)	11,469 (83.2)	14,301 (83.3)	5,052 (85.2)	10,535 (83.4)	22,497 (82.8)	45,241 (82.1)
Yes	769 (17.2)	1,651 (18.1)	2,061 (16.8)	2,714 (16.7)	815 (14.8)	1,848 (16.6)	4,359 (17.2)	9,274 (17.9)
Recent Illness								
No	3,479 (67.0)	7,343 (70.3)	10,055 (67.3)	13,119 (69.6)	4,374 (66.9)	9,372 (68.2)	20,618 (69.4)	43,011 (71.3)
Yes	1,613 (33.0)	2,842 (29.7)	4,480 (32.7)	5,352 (30.4)	1,949 (33.1)	4,138 (31.8)	8,409 (30.6)	16,104 (28.7)
Mother’s age								
15–24 year	2,235 (47.6)	4,486 (46.8)	5,229 (40.2)	7,145 (41.2)	2,269 (40.5)	4,941 (42.5)	10,870 (40.1)	23,433 (41.7)
25–34 year	2,534 (47.4)	5,091 (48.2)	8,085 (53.3)	9,990 (52.9)	3,531 (52.8)	7,380 (51.5)	15,974 (53.8)	31,530 (52.5)
35–49 year	323 (5.0)	608 (5.0)	1,221 (6.5)	1,336 (5.9)	523 (6.7)	1,189 (6.0)	2,183 (6.1)	4,152 (5.8)
Mother’s Education								
No Education	823 (14.8)	1,992 (18.8)	2,508 (15.8)	3,357 (17.5)	1,092 (15.7)	2,464 (16.1)	5,306 (17.4)	11,534 (19.0)
Primary	586 (10.8)	1,190 (11.1)	1,684 (10.6)	2,224 (11.3)	761 (11.4)	1,706 (11.4)	3,441 (11.0)	6,969 (11.1)
Secondary	2,798 (54.0)	5,458 (52.5)	7,989 (54.8)	9,858 (51.9)	3,404 (52.6)	7,403 (54.9)	15,637 (53.1)	31,674 (52.5)
Higher	885 (20.4)	1,545 (17.6)	2,354 (18.8)	3,032 (19.3)	1,066 (20.3)	1,937 (17.6)	4,643 (18.5)	8,938 (17.4)
Mother’s Exposure to mass media								
No	2,434 (44.4)	5,316 (49.9)	6,985 (44.7)	8,859 (45.1)	2,934 (44.1)	6,639 (44.4)	14,335 (46.7)	30,381 (48.9)
Yes	2,658 (55.6)	4,869 (50.1)	7,550 (55.3)	9,612 (54.9)	3,389 (55.9)	6,871 (55.6)	14,692 (53.3)	28,734 (51.1)
Residence								
Urban	1,109 (28.8)	1,977 (25.3)	3,091 (28.0)	4,003 (28.7)	1,393 (28.6)	2,996 (30.1)	6,079 (28.1)	12,111 (26.9)
Rural	3,983 (71.2)	8,208 (74.7)	11,444 (72.0)	14,468 (71.3)	4,930 (71.4)	10,514 (69.9)	22,948 (71.9)	47,004 (73.1)
Religion								
Hindu	3,574 (77.4)	7,407 (78.4)	9,691 (75.7)	13,434 (79.2)	4,170 (75.6)	7,785 (68.8)	20,940 (78.8)	43,436 (79.2)
Muslim	763 (17.8)	1,539 (17.5)	2,270 (18.7)	2,455 (15.8)	937 (18.6)	2,715 (25.2)	3,915 (16.1)	8,464 (16.4)
Christian	549 (2.9)	849 (2.1)	1,865 (3.2)	1,804 (2.6)	901 (3.4)	2,389 (4.2)	2,839 (2.5)	4,790 (2.1)
Others	206 (1.9)	390 (2.0)	709 (2.4)	778 (2.4)	315 (2.4)	621 (1.8)	1,333 (2.6)	2,425 (2.3)
Caste								
Schedule Caste	977 (24.0)	2,017 (23.6)	2,752 (24.8)	3,626 (24.1)	1,203 (25.0)	2,399 (25.8)	5,693 (24.6)	11,882 (24.2)
Schedule Tribes	1,136 (10.5)	2,174 (11.4)	3,697 (11.7)	4,030 (10.5)	1,671 (11.1)	4,108 (12.4)	6,656 (11.6)	12,185 (11.1)
Backward Caste	1,857 (46.0)	3,862 (46.0)	4,828 (42.8)	6,879 (45.6)	2,101 (43.2)	4,081 (41.8)	10,368 (43.8)	22,451 (45.6)
Others	823 (19.5)	1,587 (19.0)	2,324 (20.7)	3,035 (19.8)	981 (20.7)	1,851 (20.0)	4,725 (20.0)	9,407 (19.1)
Wealth quintile								
Poorest	1,261 (21.4)	2,744 (23.9)	3,713 (22.6)	4,665 (22.5)	1,581 (22.5)	3,870 (24.7)	7,633 (23.7)	15,492 (23.6)
Poorer	1,085 (18.6)	2,309 (21.1)	3,250 (20.3)	3,975 (18.9)	1,361 (18.5)	3,186 (21.1)	6,421 (20.1)	13,534 (21.0)
Middle	1,063 (20.7)	2,053 (20.1)	2,887 (20.4)	3,620 (19.7)	1,241 (19.8)	2,665 (20.7)	5,681 (19.8)	11,680 (20.0)
Richer	916 (20.1)	1,713 (18.2)	2,594 (19.8)	3,280 (19.8)	1,161 (20.7)	2,317 (19.4)	4,953 (18.7)	10,147 (18.8)
Richest	767 (19.2)	1,366 (16.7)	2,091 (16.9)	2,931 (19.1)	979 (18.5)	1,472 (14.1)	4,339 (17.7)	8,262 (16.6)
ICDS Utilization								
No	2,891 (70.5)	5,614 (70.6)	8,052 (65.4)	11,093 (72.9)	3,680 (69.1)	7,236 (62.6)	16,390 (68.7)	32,959 (69.5)
Yes	1,027 (29.5)	2,168 (29.4)	3,481 (34.6)	3,639 (27.1)	1,360 (30.9)	3,244 (37.4)	6,572 (31.3)	13,206 (30.5)
Region								
South	799 (20.8)	1,309 (16.5)	2,283 (21.6)	2,558 (19.1)	961 (21.2)	2,838 (29.1)	3,762 (17.8)	7,829 (17.4)
North	912 (12.9)	1,804 (13.0)	2,377 (11.2)	3,534 (14.1)	1,112 (12.6)	1,431 (6.1)	5,139 (12.5)	10,420 (12.6)
Central	975 (20.4)	2,612 (27.6)	2,579 (18.0)	4,038 (23.3)	1,029 (16.4)	1,753 (12.8)	6,438 (22.9)	14,697 (26.5)
East	1,009 (27.8)	1,989 (26.3)	3,196 (34.6)	3,922 (29.0)	1,451 (36.5)	3,015 (37.5)	6,333 (30.8)	11,586 (26.6)
West	437 (13.5)	890 (12.6)	1,039 (9.9)	1,444 (11.0)	404 (9.3)	716 (8.7)	2,578 (12.3)	5,577 (13.1)
Northeast	960 (4.6)	1,581 (4.0)	3,061 (4.7)	2,975 (3.5)	1,366 (4.0)	3,757 (5.8)	4,777 (3.7)	9,006 (3.8)
Observations	5,092	10,185	14,535	18,471	6,323	13,510	29,027	59,115

*n – frequency*,* % - weighted percentage.*

### Prevalence of complementary feeding practices

The estimated proportion of children age 6–8 months who received timely introduction of complementary foods was 49.4% (95% CI: 48.0–50.7) (Table 2). Among children age 6–23 months, 23.5% (95% CI: 22.9–24.0) met the minimum dietary diversity, 33.9% (95% CI: 33.4–34.6) met the minimum meal frequency, and 10.1% (95% CI: 9.7–10.5) had a minimum acceptable diet. Egg or flesh food consumption was 21.9% (95% CI: 21.4–22.5), whereas 48.1% (95% CI: 47.6–48.7) consumed vegetables or fruits the previous day. Complementary feeding practices consistently improved as the child’s age increased.

**Table 2:** Proportion of children who consumed appropriate complementary food in India, NFHS 2019–21

Complementary feeding practices	Age (in months)	Proportion (95% CI)
Introduction of solid, semisolid or soft foods (ISSSF)	6–8	49.4 (48.0–50.7)
	6	36.8 (34.4–39.3)
	7	50.9 (48.6–53.3)
	8	62.1 (59.8–64.3)
Minimum dietary diversity (MDD)	6–23	23.5 (22.9–24.0)
	6–11	13.2 (12.6–13.9)
	12–17	25.9 (25.0–26.7)
	18–23	32.3 (31.3–33.3)
Minimum meal frequency (MMF)	6–23	33.9 (33.4–34.6)
	6–11	27.9 (27.0–28.9)
	12–17	34.5 (33.6–35.4)
	18–23	40.2 (39.1–41.3)
Minimum acceptable diet (MAD)	6–23	10.1 (9.7–10.5)
	6–11	5.7 (5.2–6.1)
	12–17	12.1 (11.5–12.8)
	18–23	12.8 (12.1–13.5)
Egg and/or flesh food consumption (EFF)	6–23	21.9 (21.4–22.5)
	6–11	12.1 (11.4–12.8)
	12–17	24.1 (23.2–24.9)
	18–23	30.7 (29.7–31.7)
Vegetables or fruits consumption (VFC)	6–23	48.1 (47.6–48.7)
	6–11	30.4 (29.5–31.3)
	12–17	53.4 (52.5–54.4)
	18–23	62.2 (61.2–63.2)

### Association between ICDS utilization and complementary feeding practices

No significant association is found between ICDS utilization and ISSSF (aPR: 0.97, 95% CI: 0.91–1.03) (Table 3). Children age 6 to 23 months who utilized ICDS in the last 12 months had a 19% higher probability of consuming a diverse diet (95% CI: 1.13–1.25) of consuming 5 out of 8 food groups in the previous 24 h. MMF among ICDS beneficiaries is 14% lower (95% CI: 0.86–0.90) than non-beneficiaries. ICDS utilization is not significantly associated (aPR: 1.05 (95% CI: 0.96–1.15) with MAD with overall coverage remaining low. Consumption of egg and flesh foods is 27% (95% CI: 1.20–1.34) higher among children utilizing ICDS. Consumption of vegetables and fruits is 3% higher (95% CI: 1.00–1.06) among children utilizing ICDS.

**Table 3:** Association between ICDS utilization and complementary feeding practices among children age 6–23 months in India

Variables	ISSSF			MDD			MMF			MAD			EFF			VFC	
	(*n* = 6,894)^a^			(*n* = 41,023)^b^													
	aPR	95% CI	aPR		95% CI	aPR		95% CI	aPR		95% CI	aPR		95% CI	aPR		95% CI
ICDS Utilization (ref: No)																	
Yes	0.97	(0.91–1.04)	1.19**		(1.13–1.25)	0.86**		(0.83–0.90)	1.05		(0.96–1.15)	1.27**		(1.20–1.34)	1.03*		(1.00–1.06)
Child’s age (ref: 6 month)																	
7 months	1.43**	(1.32–1.55)															
8 months	1.71**	(1.58–1.84)															
Child’s age group (ref: 6–11 months)																	
12–17 months			1.95**		(1.83–2.09)	1.25**		(1.20–1.31)	2.31**		(2.08–2.57)	2.04**		(1.89–2.19)	1.75**		(1.68–1.82)
18–23 months			2.40**		(2.25–2.56)	1.47**		(1.40–1.54)	2.42**		(2.17–2.70)	2.48**		(2.31–2.66)	2.00**		(1.92–2.08)
Child’s sex (ref: Male)																	
Female	1.02	(0.97–1.08)	1.02		(0.97–1.06)	1.01		(0.97–1.05)	1.03		(0.95–1.10)	1.03		(0.99–1.08)	1.02		(0.99–1.04)
Low birth weight (ref: No)																	
Yes	0.99	(0.91–1.07)	0.96		(0.90–1.02)	0.92**		(0.88–0.97)	0.84**		(0.75–0.93)	0.95		(0.89–1.02)	0.97		(0.94–1.01)
Recent Illness (ref: No)																	
Yes	1.17**	(1.11–1.25)	1.14**		(1.09–1.20)	1.09**		(1.05–1.13)	1.19**		(1.10–1.29)	1.05		(1.00–1.11)	1.08**		(1.05–1.11)
Mother’s age (ref: 15–24 years)																	
25–34 years	0.99	(0.93–1.05)	1.08**		(1.03–1.13)	1.00		(0.96–1.04)	1.06		(0.98–1.14)	0.99		(0.95–1.04)	1.05**		(1.03–1.08)
35–49 years	1.04	(0.90–1.21)	1.20**		(1.09–1.32)	1.01		(0.93–1.10)	1.14		(0.96–1.35)	1.04		(0.94–1.16)	1.12**		(1.06–1.18)
Mother’s Education (ref: No education)																	
Primary	1.16*	(1.03–1.31)	1.17**		(1.06–1.28)	1.09*		(1.02–1.17)	1.22*		(1.04–1.42)	1.18**		(1.08–1.29)	1.09**		(1.04–1.14)
Secondary	1.15**	(1.04–1.27)	1.26**		(1.17–1.36)	1.02		(0.96–1.08)	1.16*		(1.03–1.32)	1.16**		(1.08–1.26)	1.14**		(1.09–1.18)
Higher	1.26**	(1.12–1.42)	1.34**		(1.22–1.47)	1.06		(0.99–1.14)	1.32**		(1.13–1.54)	1.27**		(1.15–1.41)	1.19**		(1.13–1.25)
Mother’s Exposure to mass media (ref: No)																	
Yes	1.14**	(1.06–1.22)	1.08**		(1.02–1.14)	1.15**		(1.10–1.20)	1.16**		(1.06–1.27)	1.14**		(1.08–1.21)	1.07**		(1.04–1.10)
Residence (ref: Urban)																	
Rural	0.91*	(0.84–0.99)	1.01		(0.94–1.08)	1.03		(0.97–1.09)	1.05		(0.94–1.18)	0.89**		(0.82–0.95)	1.00		(0.96–1.03)
Religion (ref: Hindu)																	
Muslim	1.07	(0.97–1.17)	1.26**		(1.17–1.35)	0.95		(0.89–1.01)	1.24**		(1.09–1.40)	1.77**		(1.65–1.90)	1.00		(0.96–1.05)
Christian	1.13	(0.94–1.35)	1.26**		(1.12–1.42)	1.15*		(1.03–1.30)	1.33**		(1.09–1.62)	1.39**		(1.25–1.53)	1.14**		(1.05–1.23)
Others	0.91	(0.73–1.12)	1.10		(0.96–1.27)	0.97		(0.86–1.09)	1.07		(0.84–1.36)	1.09		(0.92–1.31)	1.09*		(1.01–1.17)
Caste (ref: Scheduled caste)																	
Scheduled tribes	0.98	(0.89–1.07)	1.08		(1.00–1.17)	1.01		(0.95–1.08)	1.05		(0.92–1.19)	1.04		(0.96–1.13)	1.08**		(1.03–1.13)
Other backward castes	0.96	(0.89–1.04)	0.88**		(0.83–0.94)	0.99		(0.94–1.04)	0.87**		(0.79–0.96)	0.77**		(0.72–0.83)	0.95**		(0.92–0.99)
Others	0.99	(0.90–1.09)	0.98		(0.91–1.06)	1.01		(0.94–1.07)	0.93		(0.81–1.06)	0.89*		(0.82–0.98)	0.99		(0.95–1.03)
Wealth Quintile (ref: Poorest)																	
Poorer	0.88*	(0.79–0.97)	1.03		(0.96–1.11)	0.93*		(0.88–0.99)	0.96		(0.84–1.08)	0.94		(0.88–1.01)	0.95*		(0.91–0.99)
Middle	1.00	(0.90–1.10)	1.05		(0.97–1.14)	1.02		(0.96–1.09)	1.08		(0.94–1.24)	0.87**		(0.80–0.95)	0.99		(0.95–1.03)
Richer	1.02	(0.92–1.15)	1.14**		(1.05–1.25)	1.09*		(1.01–1.17)	1.25**		(1.08–1.44)	0.91*		(0.83–1.00)	1.02		(0.97–1.07)
Richest	1.04	(0.92–1.19)	1.15*		(1.03–1.28)	1.17**		(1.07–1.27)	1.31**		(1.10–1.56)	0.85**		(0.76–0.96)	1.05		(0.99–1.12)
Region (ref: South)																	
North	0.86**	(0.78–0.95)	0.75**		(0.69–0.82)	1.05		(0.98–1.12)	0.92		(0.81–1.04)	0.26**		(0.24–0.29)	1.00		(0.96–1.05)
Central	0.70**	(0.63–0.77)	0.63**		(0.58–0.67)	0.88**		(0.83–0.94)	0.62**		(0.55–0.70)	0.31**		(0.28–0.33)	0.90**		(0.87–0.95)
East	0.97	(0.89–1.07)	1.25**		(1.16–1.34)	1.14**		(1.07–1.22)	1.49**		(1.32–1.68)	0.87**		(0.81–0.93)	1.26**		(1.21–1.32)
West	0.90*	(0.81–1.00)	0.64**		(0.57–0.72)	0.81**		(0.75–0.89)	0.64**		(0.53–0.77)	0.34**		(0.30–0.39)	0.95		(0.90–1.01)
Northeast	1.03	(0.90–1.19)	1.07		(0.98–1.18)	0.92		(0.84–1.01)	1.08		(0.92–1.27)	0.86**		(0.79–0.95)	0.98		(0.92–1.04)

** *p* < 0.005, * *p* < 0.01

*aPR* adjusted Prevalence ratio, *CI *confidence interval

^a^ Data missing for 3291 observations

^b^ Data were missing for 18,092 observations

Among the covariates, child’s age and recent illness were positively associated with all six complementary feeding indicators except for recent illness with EFC (Table 3). Mothers’ education and mass media exposure are positively associated with all outcomes except for MMF. Mother’s age is positively associated with MDD and VFC. Compared with the Hindu religion, Muslims and Christians are more positively associated with MDD, MAD and EFF.

## Discussion

This study assessed the association between the ICDS utilization and six complementary feeding practices indicators in India. The results show that ICDS utilization is positively associated with improved complementary feeding practices such as diet diversity, egg & flesh food consumption, and consumption of vegetables and fruits. Further, child’s age, mother’s media exposure and education, religion are significantly associated with complementary feeding practices.

Our findings are consistent with matching analysis of India’s NFHS-4 data by Petrovika (2021) which reported that regular participation in ICDS activities including receipt of supplementary food, health checkups, growth monitoring and regular attendance were linked with better adherence to recommended complementary practices, particularly diverse diets, animal source food consumption, and vegetable & fruit consumption [22].

Among covariates, mothers’ education is significantly linked with all six complementary feeding indicators, which is consistent with previous findings from DHS data analysis of six South Asian countries and from a recent study in India [17, 23]. Educated mothers are more likely to understand the importance of nutrition and appropriate feeding practices, which can lead to improved complementary feeding practices. Similar to an existing study, mass media exposure is significantly associated with improved complementary feeding [18].

### Introduction to semi-solid foods

Supplementary nutrition has been the key focus of ICDS and it is provided to beneficiaries 300 days in a year either as take-home rations (THRs) or Hot cooked meal (HCM). In states such as Tamil Nadu and Kerala, THR is distributed as fortified, premixed packets, whereas in other states, it is often supplied as raw commodities. Supplementary nutrition serves as a key entry point for mothers to engage with other components of the ICDS. By delivering energy-dense and protein-rich THR to young children, the ICDS ensures that age-appropriate semisolid foods are physically available when infants are developmentally ready to start complementary feeding. Our analysis revealed that ICDS utilization is not associated with timely introduction to semi-solid foods at 6–8 months, consistent with the findings by Petrikova (2021) [22]. This indicates that supplementary nutrition provided through ICDS may have very minimal influence on promoting timely introduction of semi-solid foods, with less than half the infants in this age group meeting their requirement. Delaying the introduction of complementary foods beyond 6 months can lead to growth faltering and increase the risk of undernutrition [24]. While each state in India can formulate their THR according to specific nutritional needs and local contexts, regular distribution along with counseling and home visits by Anganwadi workers can encourage families to initiate complementary feeding at the recommended age of 6 months and to use the provided rations as safe, nutritious, first semisolid food [25].

### Diet diversity and adequacy

A descriptive trend by Agarwal et al. (2024) suggests that states performing better on ICDS also achieve improved outcomes in MDD, MMF, and MAD [26]. In contrast, our study found that ICDS utilization was associated with improvements only in dietary diversity. Surprisingly, ICDS utilization is negatively associated with child’s feeding frequency. MAD is an essential indicator capturing information of both diet diversity and meal frequency reflecting an integrated measure for complementary feeding practices. A comprehensive study in Rwanda involving 241 children aged 6–23 months provided robust clinical evidence that children meeting MAD have significantly lower stunting risk [27]. Dixit et al. (2018) reported that children under 3 years of age who utilized ICDS services were more likely to be stunted, wasted or underweight, suggesting a shift from supplementary feeding to improved environmental hygiene and child feeding practices [15]. At the outset, our findings indicated that ICDS had no significant influence on achieving the MAD. Growing consumption of junk foods and sugary beverages combined with food insecurity could have displaced the recommended meal frequency and diluted the positive effects of dietary diversity, which requires further investigation. An exploratory participative research study in Egypt indicates that consumption of junk foods and other non-nutritive liquids among infants and young children are pushed into the children’s daily diets, often replacing necessary calories and nutrients that should come from adequate, frequent, and diverse complementary meal [28]. A qualitative study on IYCF practices among caregivers of children under 2 years in Mumbai emphasizes preventing the early introduction of sugary and non-nutritious processed foods, and empower mothers to confidently follow the recommended IYCF guidelines, with the risk likely to be greater in the poorest communities, where inexpensive snack foods are widely available and marketed [29].

Our analysis revealed that the proportions of children aged 6–23 months from India who achieved MDD, MMF and MAD were 23.5%, 34.0%, and 10.1%, respectively. In comparison, pooled analysis data from 80 LMICs revealed higher rates: 27.1% for MDD, 49.7% for MMF, and 17.3% for MAD [30]. This highlights that India lags behind the average for LMICs with only one in ten children receiving both a sufficiently diverse diet and meal frequency.

### Egg and flesh food consumption

Egg and flesh food consumption during complementary feeding is highly important for improving child health outcomes. Controlled trials have demonstrated that early egg introduction improved growth in young children [31, 32]. Our analysis revealed that young children utilizing any ICDS benefits were 27% more likely to consume egg and/or flesh food. Compared with children from Hindu households, Muslims and Christians have a significantly greater chance of consuming EFF, which may be due to religious dietary norms and cultural practices. Several states in India provide eggs for young children under the ICDS to help meet their nutritional requirements and improve the quality of protein in their diet. In Andhra Pradesh, the YSR Sampoorna Poshana Plus Scheme provides 30 eggs per month for children aged 6–36 months for beneficiaries in scheduled and tribal subplan mandals [25].

### Vegetable and fruits consumption

Evidence suggests that increased consumption of vegetables and fruits reduces the risk for many chronic diseases and cancer [33]. Our analysis revealed that one in two children in India have not consumed any vegetables or fruits, with children of mothers with higher education; mass media exposure has a greater likelihood of VFC, similar to the findings of a study by Allen et al. on global estimates and trends of zero vegetable and fruit consumption [34]. The global estimate also reveals that while there was a significant decrease in zero vegetable and fruit consumption in India, it remains highest in the Southeast Asian and Western Pacific regions, with over 56% of children aged 6–23 months not consuming vegetables or fruits. A study from the UK (1992) reported that feeding fruits and vegetables in home early at 6 months increased acceptance at seven years of age [35]. A review by Mennella et al. reported that flavors in the maternal diet are transmitted via amniotic fluid and breast milk; thus, early maternal exposure familiarizes infants with the flavors of vegetables and fruits, increasing their acceptance [36]. A study involving 44 farm women in rural India found that introducing kitchen gardens increased their consumption of green leafy vegetables and fruits [37]. Caregiver modeling—where caregivers themselves eat fruits and vegetables—can positively influence infants’ acceptance. NutriGardens under ICDS can be a practical, community-based strategy to increase fruit and vegetable consumption, enhancing child nutrition and health.

### Barriers to ICDS utilization and complementary feeding practices

Barriers to the utilization and implementation of the ICDS could be considered from the user perspective, at the field staff level and at the program level. Recent evidence indicates that the utilization of any ICDS services among children aged 6–59 months improved from 58% in 2015–2016 to 71% in 2019–2021 in India [10]. Our study shows that only 30.5 children age 6–23 months utilized all three components from ICDS – receipt of take-home rations once a week, weight measured once a month and health checkup at least once a month at the Anganwadi centre. A geospatial analysis indicated wide inter-district variations in utilization of ICDS, with rural and poor households positively associated, and low maternal education negatively associated, with ICDS utilization [38]. A study reports that higher coverage of take-home rations (THR) in Tamil Nadu is above the national average which provides fortified premix. Most beneficiaries were satisfied with taste, consistency and ease of preparation, however, 83% reported sharing of rations within the household and inconsistent quality and distribution may dilute its effectiveness [39]. A recent review by Kaur et al. (2025) identified several key barriers to optimal complementary feeding in India, including socioeconomic constraints, cultural beliefs and food taboos, inadequate health system support, poor access to nutrition services, a lack of family and community support, and food insecurity. The review also highlighted key gaps in public health interventions, including inequitable allocation of resources, with high-burden states often underserved; inadequate training and support for Anganwadi workers; and limited effective counseling and behavior change communication [40]. A study conducted in Jaipur, India by Nagar and Talikoti (2022) assessed knowledge of complementary feeding among mothers registered at Anganwadi centers and reported gaps in knowledge and practice but reinforced the role of the ICDS in providing supplementary nutrition and influencing feeding behaviors [41]. AWWs play a vital role in promoting IYCF practices in the community. A study by Chaturvedi et al. (2014) revealed that the adoption of appropriate IYCF practices is strongly influenced by AWWs’ level of knowledge and skills. Caregivers visiting AWWs with superior IYCF knowledge have demonstrated greater knowledge of IYCF practices [42].

Similar to India’s ambitious ICDS, many LMICs have large-scale early childhood and nutrition programs. Notably, Bangladesh’s Shishu Bikash Kendra (Child Development Centers); Nepal’s Early Childhood Development Centers; and South Africa’s Integrated Nutrition Programme. Programs such as Alive & Thrive operate in multiple LMICs, supporting governments in scaling up nutrition interventions, including complementary feeding through health systems and community platforms.

Evidence from multiple countries shows that prioritizing complementary feeding involving multiple stakeholders including health services, applying behavioral change principles, motivating frontline workers and leverage mass media and community support lead to higher rates of timely and appropriate complementary feeding [43]. While supplementary nutrition and growth monitoring are prioritized and implemented in ICDS, behavior change communication and interpersonal communication, particularly on IYCF, are less consistently delivered and monitored, with noted gaps in frontline worker capacity and counseling quality [44]. Interpersonal communication to mothers could focus on improving mother-child interaction by encouraging practical feeding skills, responsive feeding and develop positive emotional bonding during feeding.

### Strengths and limitations

This study used nationally representative survey data to provide updated evidence on the role of the ICDS program in improving complementary feeding practices, including the consumption of eggs, flesh foods, and fruits and vegetables in India. It has few limitations. Owing to its cross-sectional design, the survey cannot establish causality between factors; it only reveals associations. There are possibilities for residual confounding after controlling for socioeconomic characteristics. Additionally, exclusion bias is possible because the analysis focuses on last-born usual resident children living with their mothers and might inadvertently omit migrant populations that the survey itself may have missed. Despite these limitations, our study reemphasizes the importance of early childhood care and development services as excellent opportunities to improve feeding practices, young children’s nutritional status and health outcomes.

## Conclusion

Despite the expansion and wide coverage of the Integrated Child Development Services in India, the programme has limited influence on improving complementary feeding practices in India. Services can incorporate more on improving maternal literacy, behavior change communication and counselling activities to enhance quality mother-child interactions. Targeted efforts to improve service delivery to young children by Integrated Child Development Services could support better complementary feeding practices.

## Data Availability

This study utilized data from a nationally representative survey that is freely available in the public domain. The dataset can be accessed through the International Institute for Population Sciences (IIPS), Mumbai, by following the standard data request procedures at [https://www.iipsdata.ac.in/data_catalog].

## Abbreviations

LMICs: Low- and middle-income countries
NFHS: National Family Health Survey
NHM: National Health Mission
ICDS: Integrated Child Development Services
AWW: Anganwadi Workers
THR: Take Home Rations
HCM: Hot cooked meal
WHO: World Health Organization
IYCF: Infant and Young Children Feeding
MoHFW: Ministry of Health and Family Welfare
IIPS: International Institute for Population Sciences
DHS: Demographic and Health Survey
aPR: adjusted Prevalence Ratio
ISSSF: Introduction of solid, semisolid or soft foods 6-8 months
MDD: Minimum dietary diversity 6–23 months
MMF: Minimum meal frequency 6–23 months
MAD: Minimum acceptable diet 6–23 months
EFF: Egg and/or flesh food consumption for 6–23 months
VFC: Vegetable or fruit consumption for 6–23 months
